# Supplementary material for: Protein sequence evolution underlies interspecies incompatibility of a cell fate determinant
Source: bioRxiv. 2025 Aug 2:2025.08.02.668269. Preprint. [Version 1] doi: 10.1101/2025.08.02.668269 (PMC12324426; doi:10.1101/2025.08.02.668269)
Supplement: Supplement 1 [file media-1.pdf]

Supplementary Material for

Protein sequence evolution underlies interspecies incompatibility of a cell fate determinant

Emily L. Rivard, John R. Srouji, Anastasia Repouliou, Cassandra G. Extavour

This document contains the following materials:

- Supplemental Figures S1 through S9
- Supplemental Tables S1 through S8
- Supplemental References

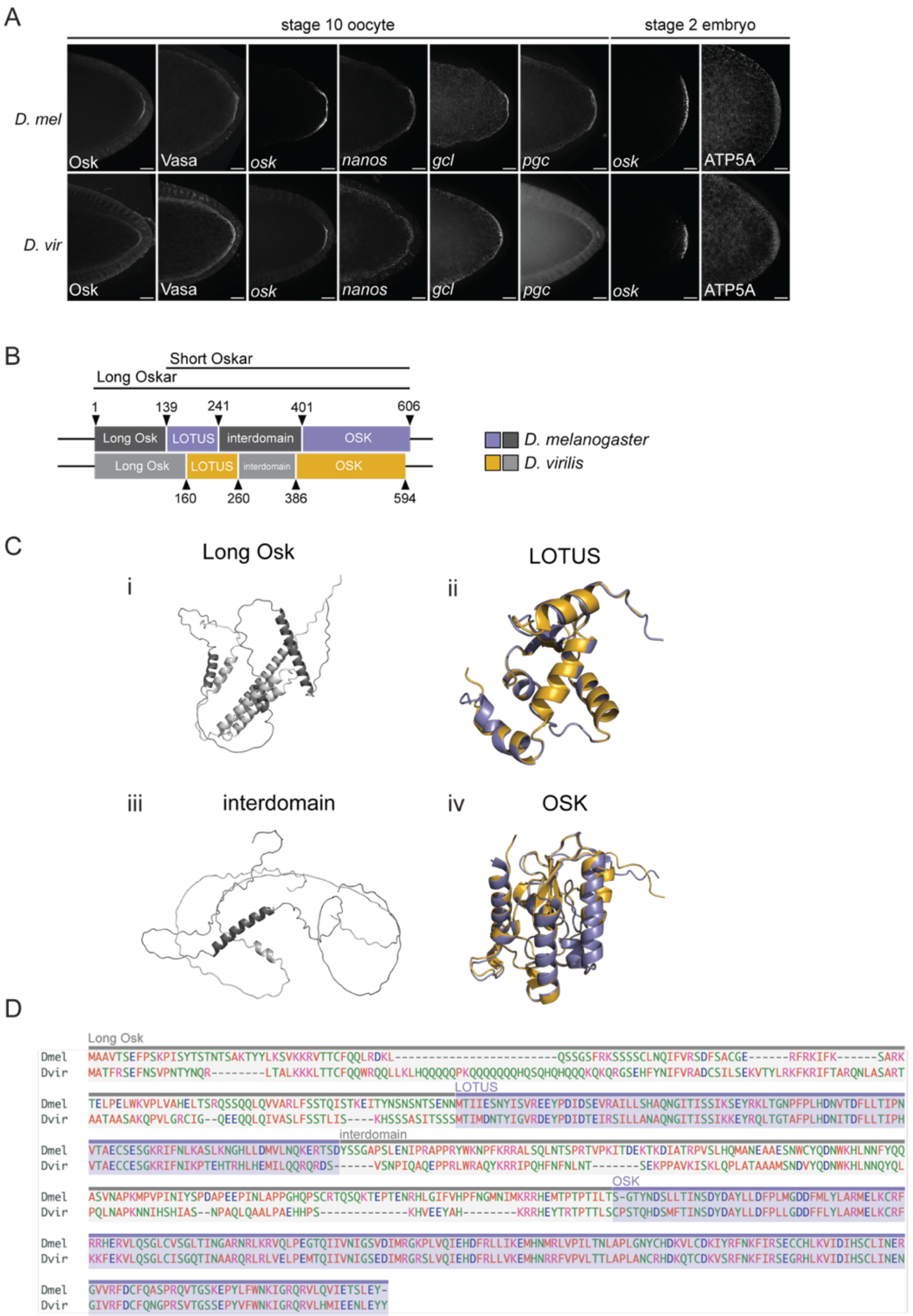

Figure S1. A) *D. melanogaster* (*D. mel*, top row) and *D. virilis* (*D. vir*, bottom row) stage 10 oocytes with Oskar, Vasa, *oskar*, *nanos*, *germ cell-less* (*gcl*), and *polar granule component* (*pgc*) labeled, and stage 2 embryos with *oskar* and mitochondria labeled (with anti-ATP5A antibody staining). All tissues are oriented with posterior to the right. Micrographs are maximum intensity projections of 20 optical sections (step size = 0.5 μm) and scale bars represent 20 μm. **B)** Schematic of *D. melanogaster* (top) and *D. virilis* (bottom) Oskar protein domain structures with amino acid residue number positions demarcating boundaries between domains. **C)** Individual *D. melanogaster* and *D. virilis* Oskar domain structural predictions generated with AlphaFold3 [1] and aligned in PyMOL (*D. melanogaster*: dark gray disordered regions, purple folded regions; *D. virilis*: light gray disordered regions, gold folded regions). **D)** Alignment of *D. melanogaster* and *D. virilis* Oskar protein sequences generated with MUSCLE. Domains shown in (**B**) are designated with colored background boxes.

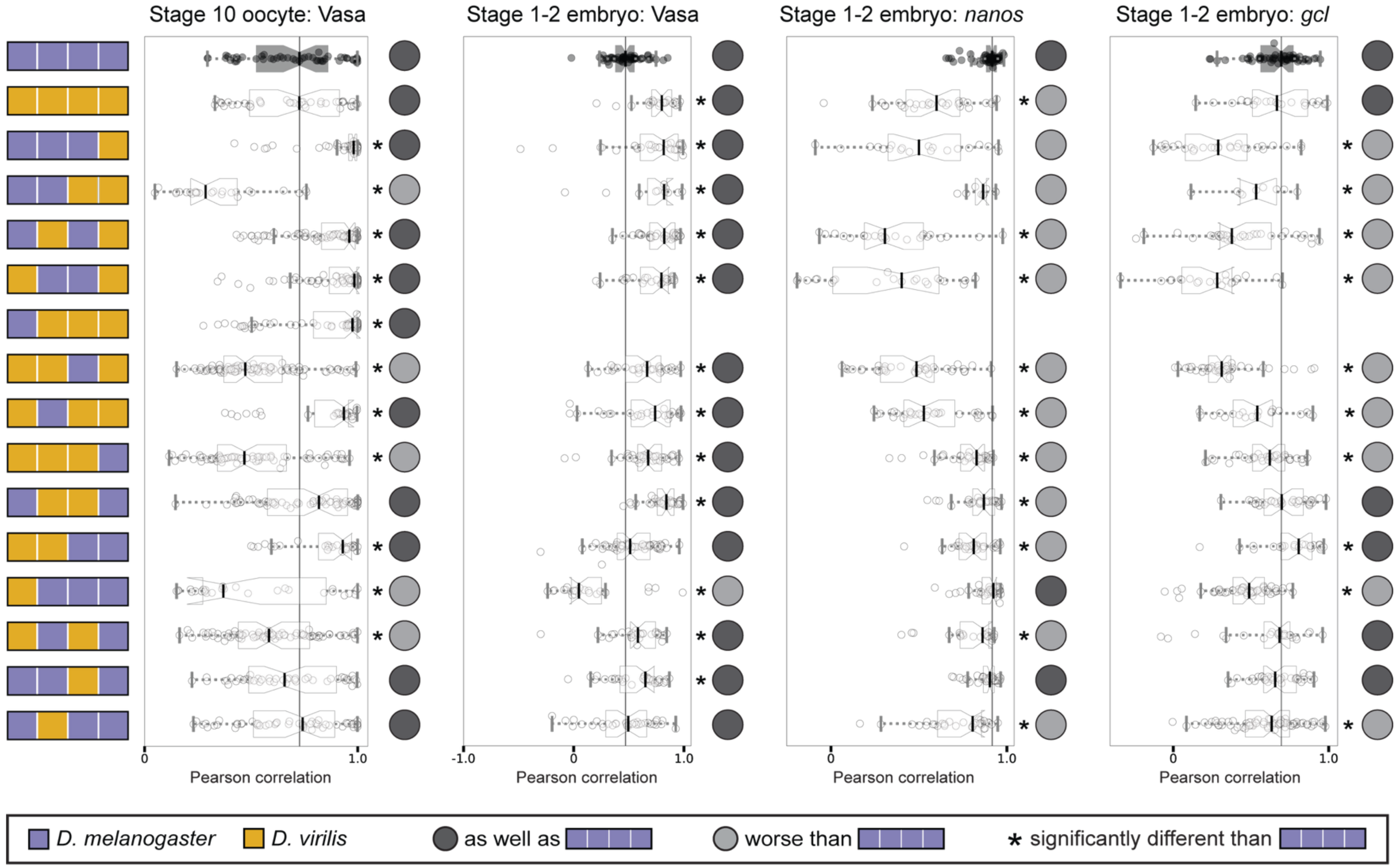

Figure S2. Pearson correlation scores between intensities of HA and Vasa, *nanos*, or *gcl* in stage 10 oocytes and stage 1–2 embryos from mothers expressing chimeric *oskar* in an *oskar* RNA null background (*y[1] sc[*] v[1]; Kr[If-1]/CyO; TI{w[+mC]=TI}osk[0.EGFP])*.. For the chimeric genotype with the Long Osk domain from *D. melanogaster* and the other three domains from *D. virilis*, sufficient posterior HA enrichment was found in oocytes to pass our threshold for analysis of germ plasm enrichment, but no embryos had sufficient enrichment to be included in the analysis. Distribution for eggs laid by *D. melanogaster* mothers expressing the *D. melanogaster oskar* chimera (positive control) is in black with the median marked with a black line. Significant difference from positive control distribution (p-value < 0.05, estimated from the distribution of difference of means between sample and control from 100,000 simulated samples) indicated by asterisk. Circles to the right of the plots indicate data interpretation as follows: dark gray = chimera enriches the germ plasm molecule at least as well as *D. melanogaster oskar*; light gray = chimera enriches the germ plasm molecule worse than *D. melanogaster oskar*.

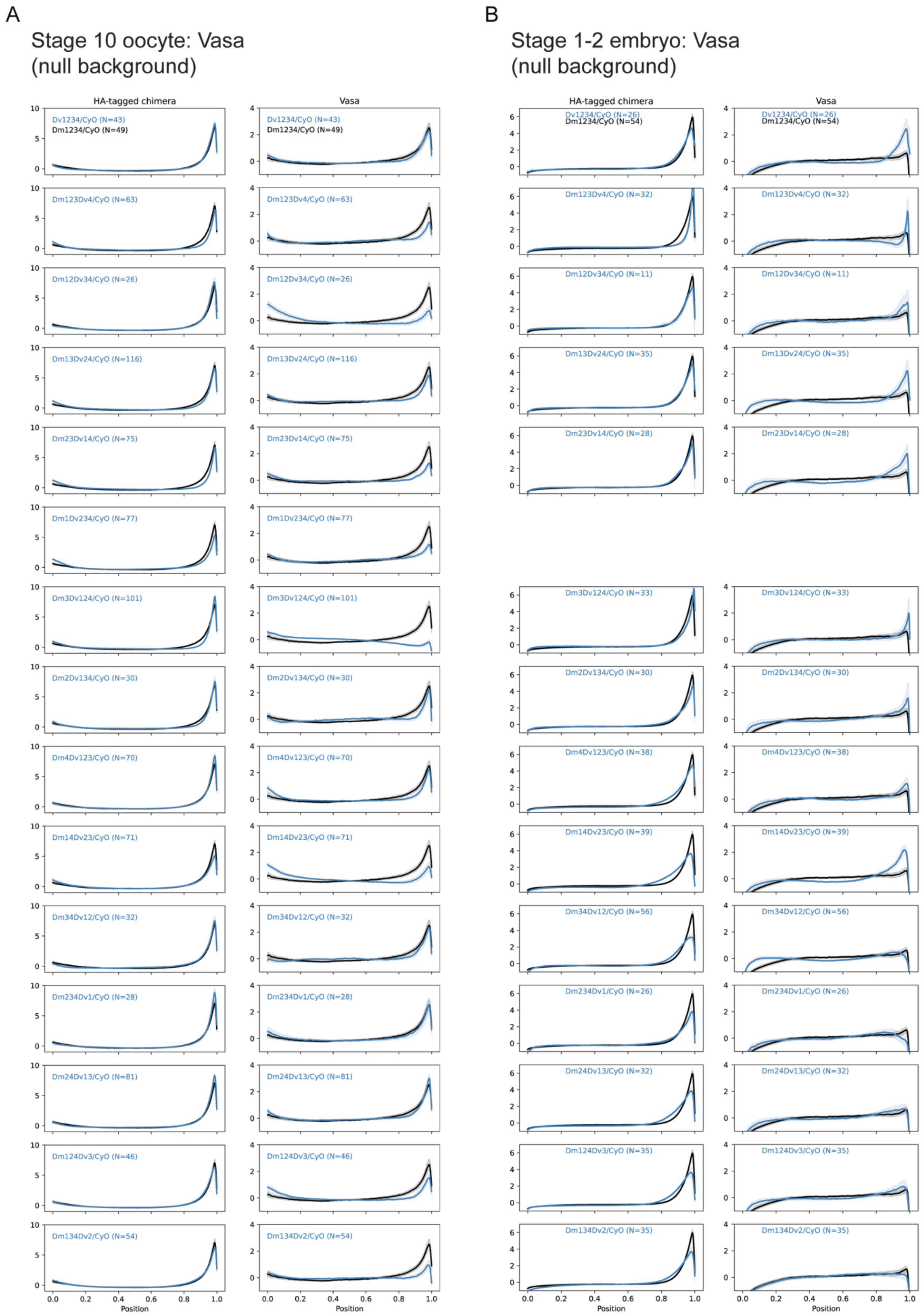

A
Stage 10 oocyte: Vasa
(null background)
B
Stage 1-2 embryo: Vasa
(null background)
HA-tagged chimera
Vasa
Dv1234/CyO (N=43)
Dm1234/CyO (N=49)
Dm123Dv4/CyO (N=63)
Dm12Dv34/CyO (N=26)
Dm13Dv24/CyO (N=116)
Dm23Dv14/CyO (N=75)
Dm1Dv234/CyO (N=77)
Dm3Dv124/CyO (N=101)
Dm2Dv134/CyO (N=30)
Dm4Dv123/CyO (N=70)
Dm14Dv23/CyO (N=71)
Dm34Dv12/CyO (N=32)
Dm234Dv1/CyO (N=28)
Dm24Dv13/CyO (N=81)
Dm124Dv3/CyO (N=46)
Dm134Dv2/CyO (N=54)
Dv1234/CyO (N=26)
Dm1234/CyO (N=54)
Dm123Dv4/CyO (N=32)
Dm12Dv34/CyO (N=11)
Dm13Dv24/CyO (N=35)
Dm23Dv14/CyO (N=28)
Dm3Dv124/CyO (N=33)
Dm2Dv134/CyO (N=30)
Dm4Dv123/CyO (N=38)
Dm14Dv23/CyO (N=39)
Dm34Dv12/CyO (N=56)
Dm234Dv1/CyO (N=26)
Dm24Dv13/CyO (N=32)
Dm124Dv3/CyO (N=35)
Dm134Dv2/CyO (N=35)
Position

C

Stage 1-2 embryo: *nanos*
(null background)

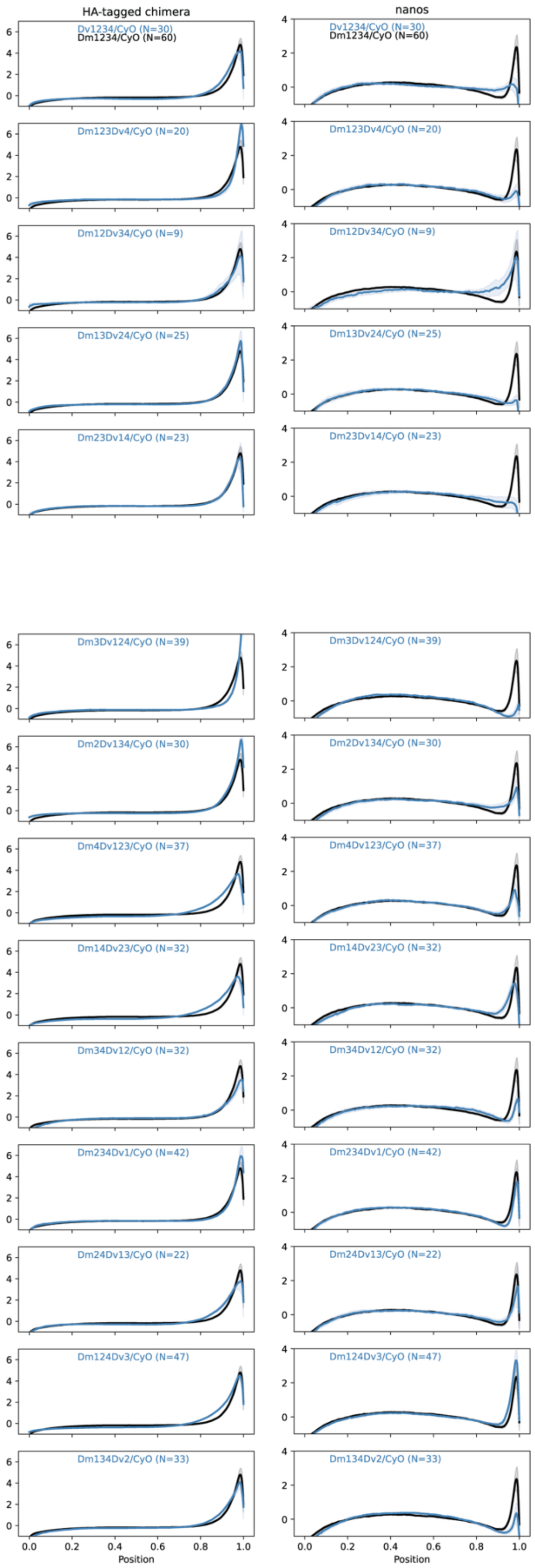

D

Stage 1-2 embryo: *gcl*
(null background)

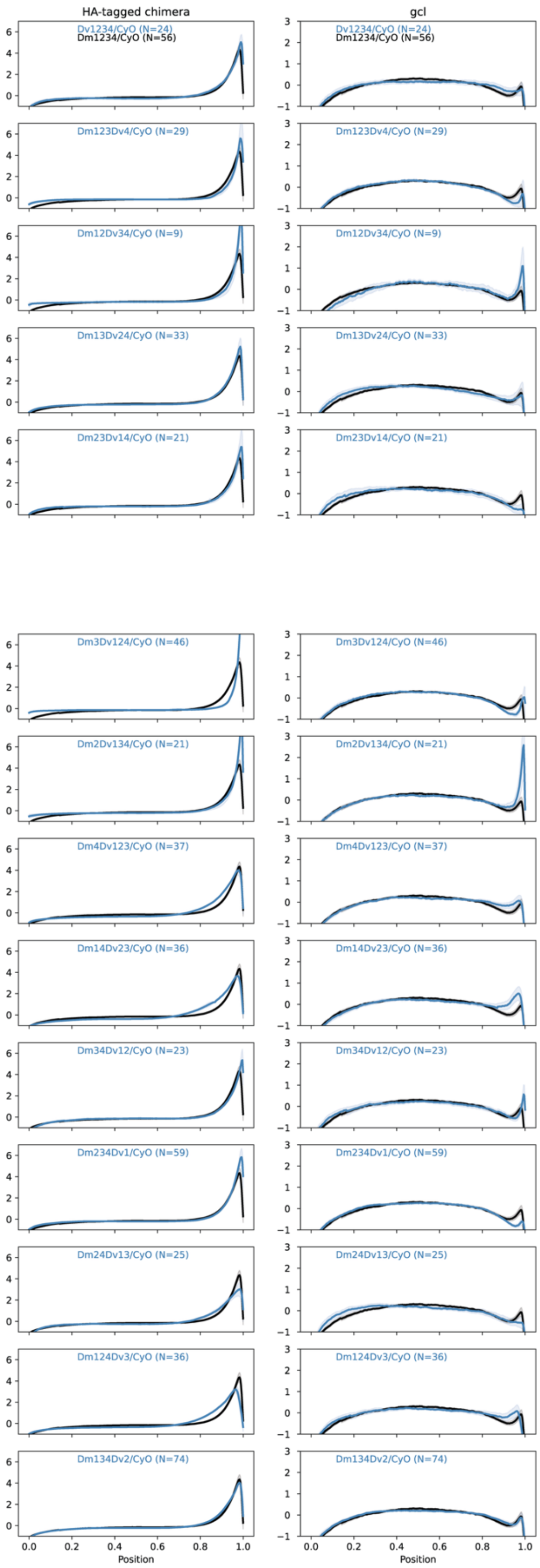

Figure S3. Vasa, *nanos,* and *gcl* signal enrichment (z-score) across stage 10 oocytes and stage 1–2 embryos in the *oskar* RNA null background *y[1] sc[*] v[1]; Kr[If-1]/CyO; TI{w[+mC]=TI}osk[0.EGFP]*. On the X axis, 0 designates the anterior and 1 is posterior. Mean (solid lines) and 95% confidence intervals (shaded regions) are shown. Black lines are measurements from positive control animals expressing the *D. melanogaster oskar* construct. Blue lines are measurements from each chimera genotype. Sample sizes for the measurements in each plot are noted in parentheses.

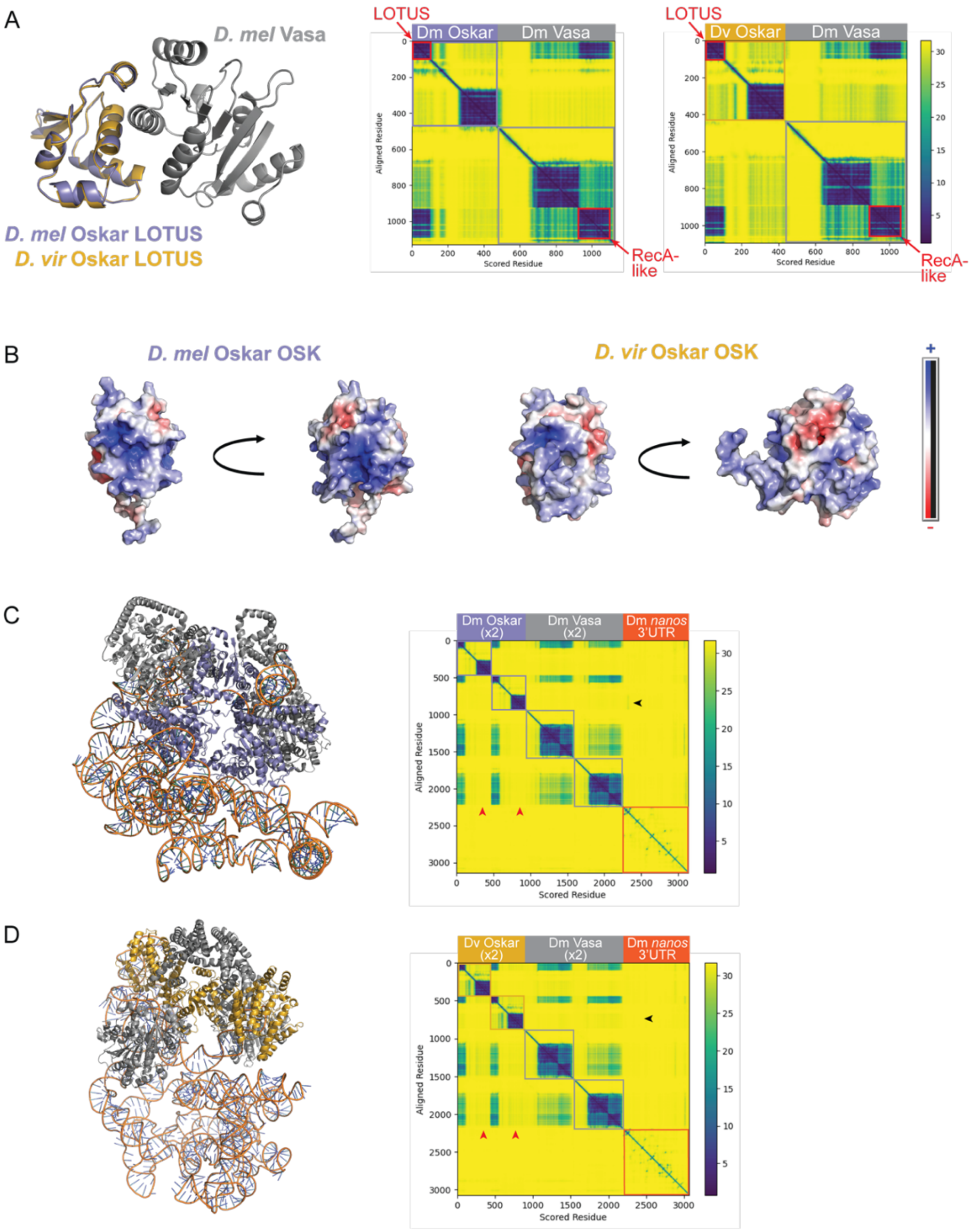

Figure S4: **A)** AlphaFold3 [1] predictions for *D. melanogaster* (purple) and *D. virilis* short Oskar interactions with *D. melanogaster* Vasa (gray). The LOTUS domains of *D. melanogaster* and *D. virilis* Oskar, as well as *D. melanogaster* Vasa, are shown in structural predictions on the left. On the right, predicted aligned error plots reflect the confidence in the relative placement of residues in the predicted structure. Blue regions mark areas with low error and therefore higher confidence. Colored boxes designate the boundaries between the different proteins in each prediction. **B)** Electrostatic maps for AlphaFold3 predictions of the OSK domain from *D. melanogaster* (left) and *D. virilis* (right) Oskar protein. Blue indicates more positive and red indicates more negative surface charge. **C, D)** Structural predictions and accompanying predicted aligned error plots for two copies of *D. melanogaster* (C) and *D. virilis* (D) short Oskar in complex with two copies of *D. melanogaster* Vasa and the *D. melanogaster nanos* 3'UTR. Black arrowheads mark where the *D. melanogaster* Oskar OSK domain has moderate predicted confidence of interaction with the *nanos* 3'UTR while *D. virilis* Oskar does not. Red arrowheads mark where the *D. virilis* Oskar OSK domain has moderate predicted confidence of interaction with Vasa while *D. melanogaster* Oskar does not.

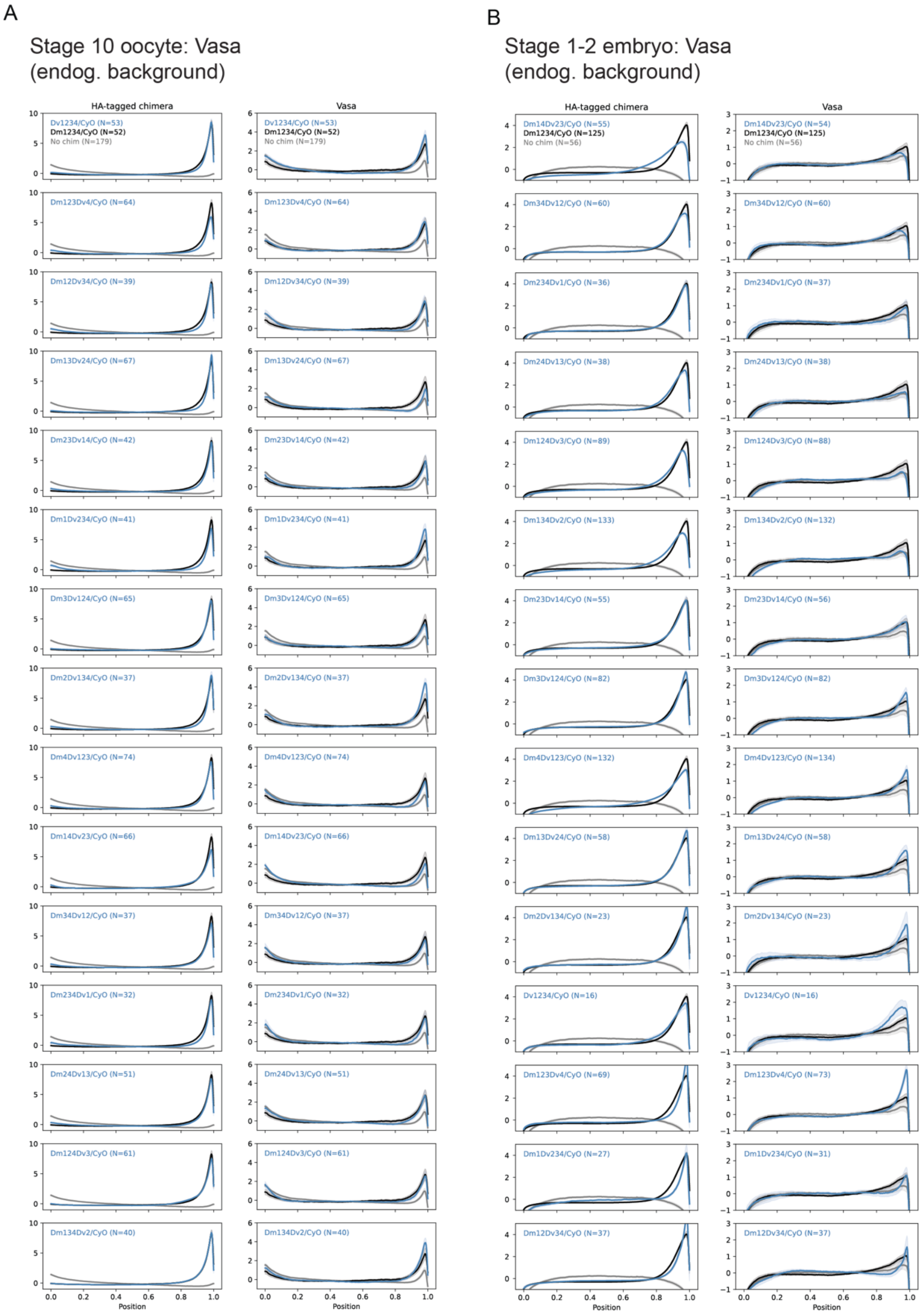
A
Stage 10 oocyte: Vasa
(endog. background)
B
Stage 1-2 embryo: Vasa
(endog. background)
HA-tagged chimera
Vasa
Dv1234/CyO (N=53)
Dm1234/CyO (N=52)
No chim (N=179)
Dm14Dv23/CyO (N=55)
Dm1234/CyO (N=125)
No chim (N=56)
Dm14Dv23/CyO (N=54)
Position

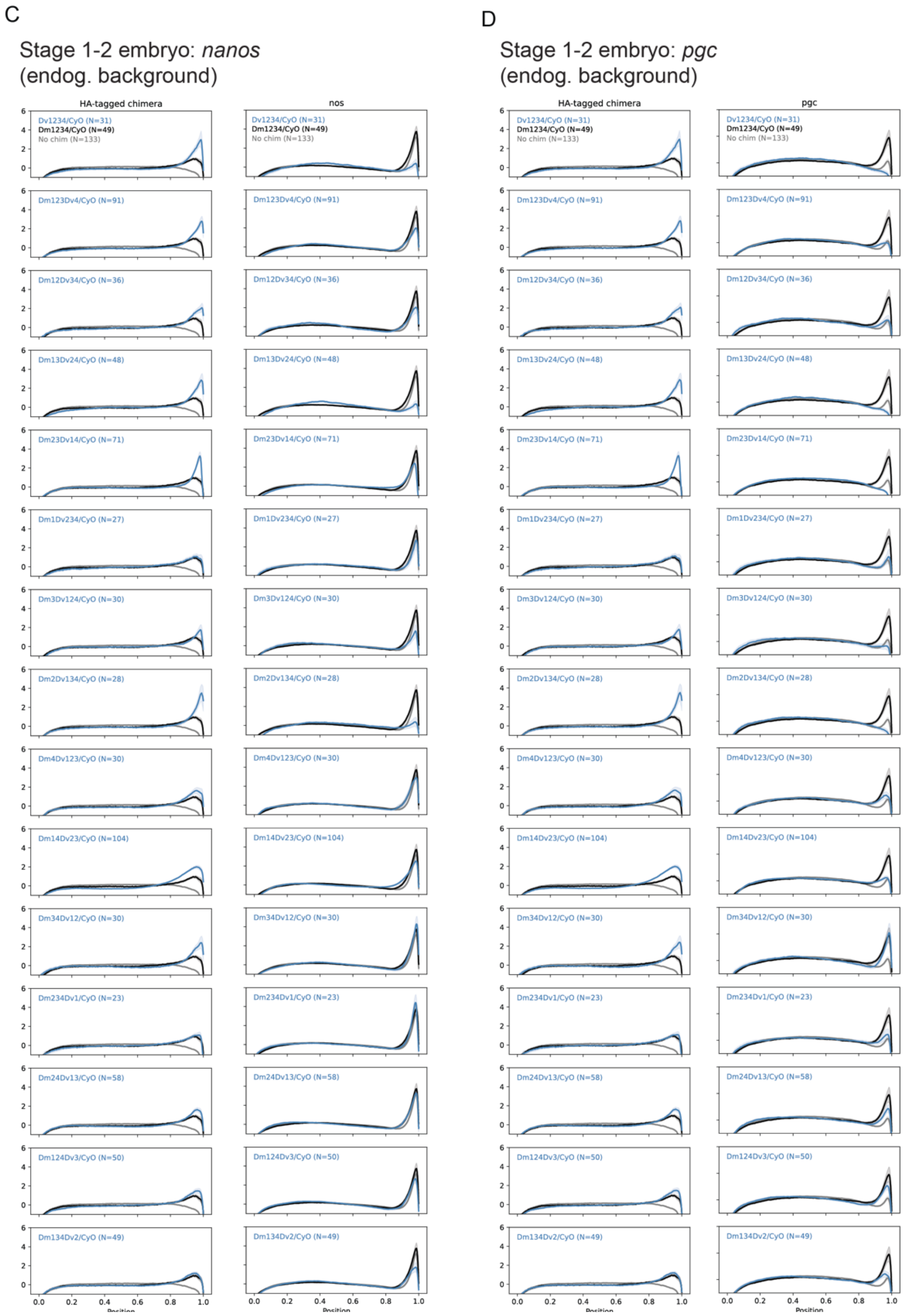

Figure S5. Vasa, *nanos,* and *pgc* signal enrichment (z-score) across stage 10 oocytes and stage 1–2 embryos for chimeras expressed in a background with endogenous *oskar*. On the X axis, 0 represents the anterior and 1 is the posterior. Mean (solid lines) and 95% confidence intervals (shaded regions) are shown. Gray lines are measurements from flies not expressing a chimeric construct (only endogenous Oskar is expressed). Black lines are measurements from positive control animals expressing the *D. melanogaster oskar* construct. Blue lines are measurements from each chimera genotype. Sample sizes for the measurements in each plot are noted in parentheses.

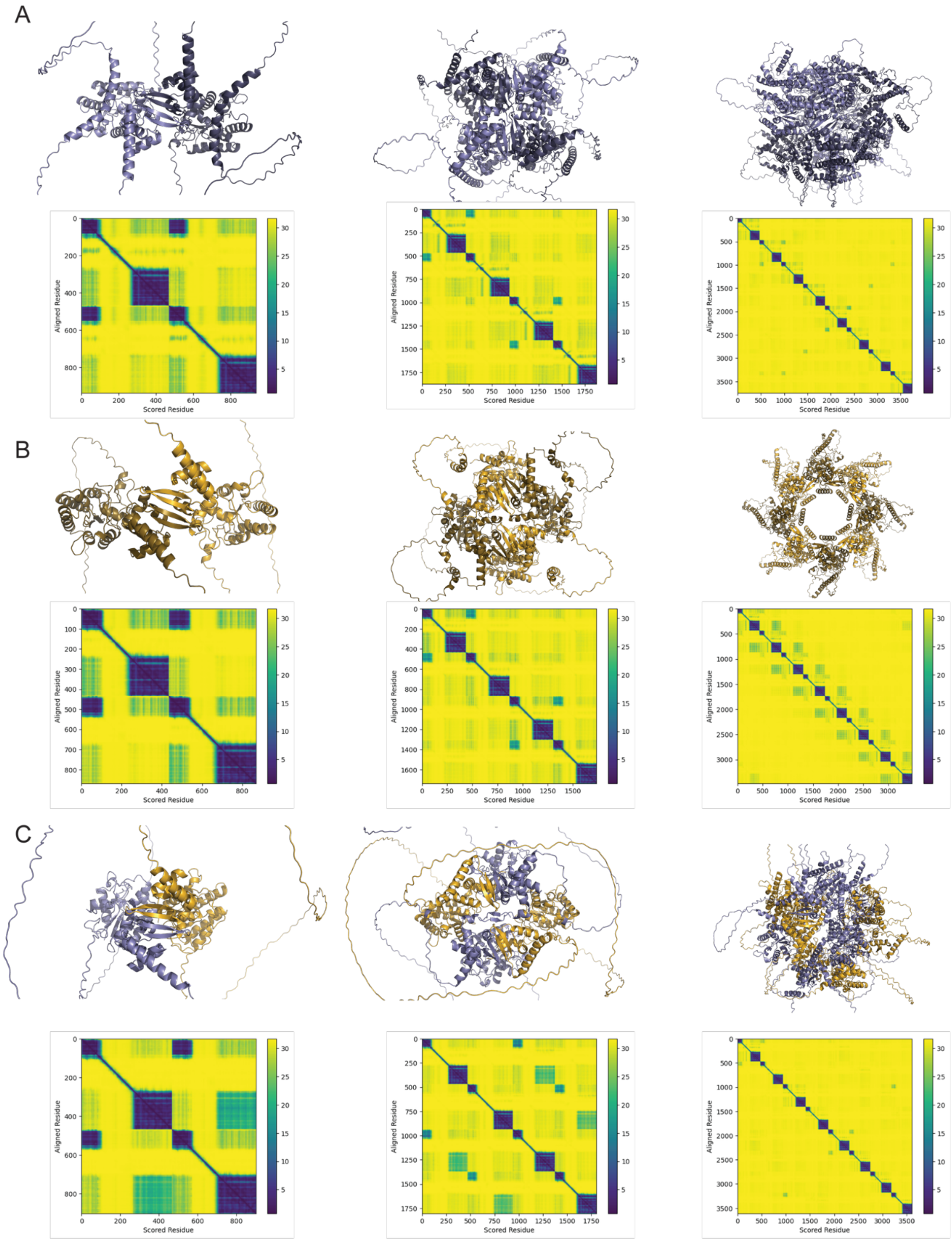
A
B
C
Aligned Residue
Scored Residue

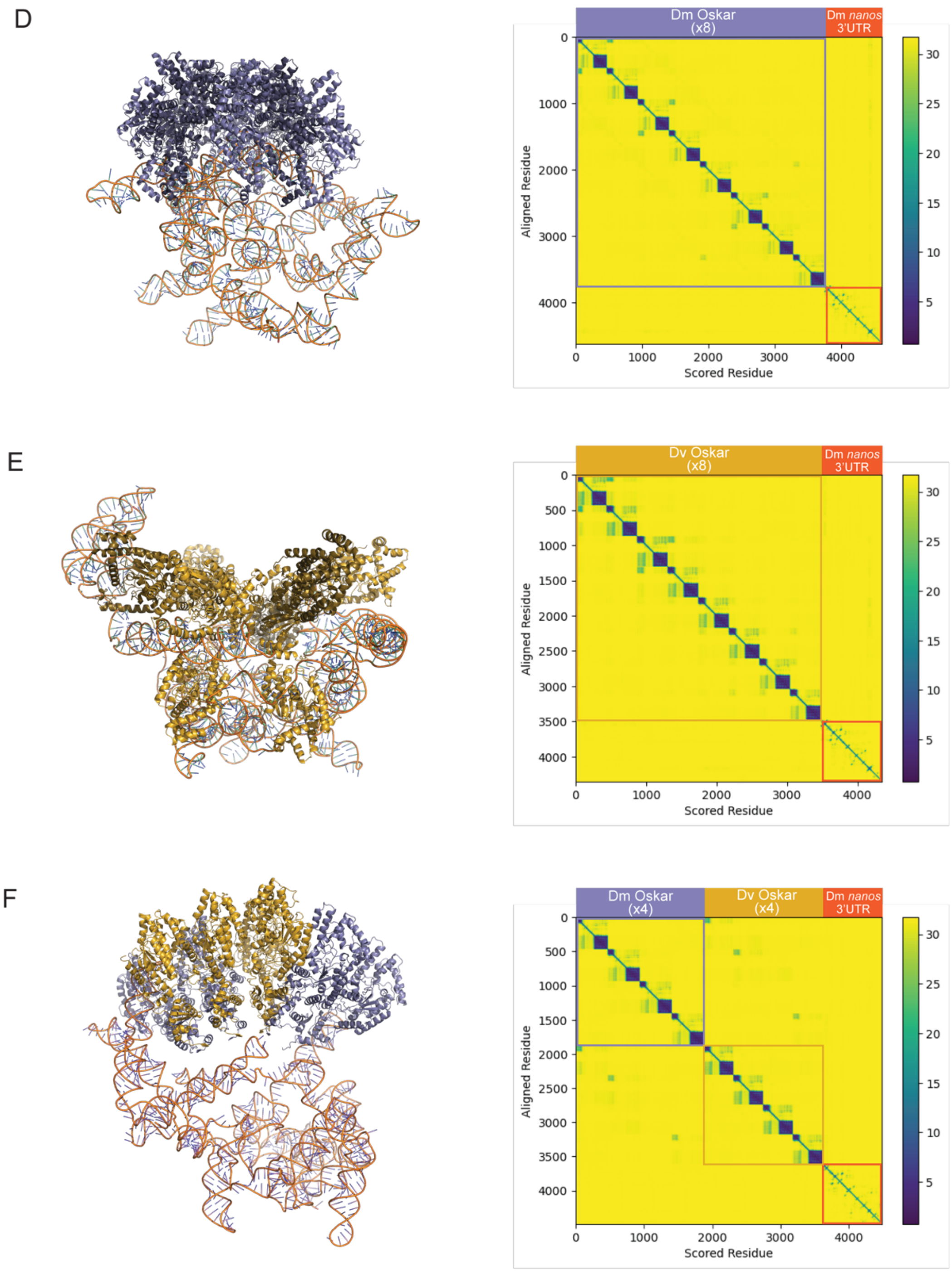

Figure S6. A–C) AlphaFold3 structural predictions and accompanying predicted aligned error plots for one (left column), two (middle column) or four (right column) monomers of each of *D. melanogaster* Short Oskar (**A**), *D. virilis* Short Oskar (**B**), both *D. melanogaster* and *D. virilis* Short Oskar (**C**). **D–F)** AlphaFold3 structural predictions and predicted aligned plots for eight copies of *D. melanogaster* (**D**) *D. virilis* (**E**), and *D. melanogaster* and *D. virilis* Short Oskar (**F**) with *D. melanogaster nanos* 3'UTR.

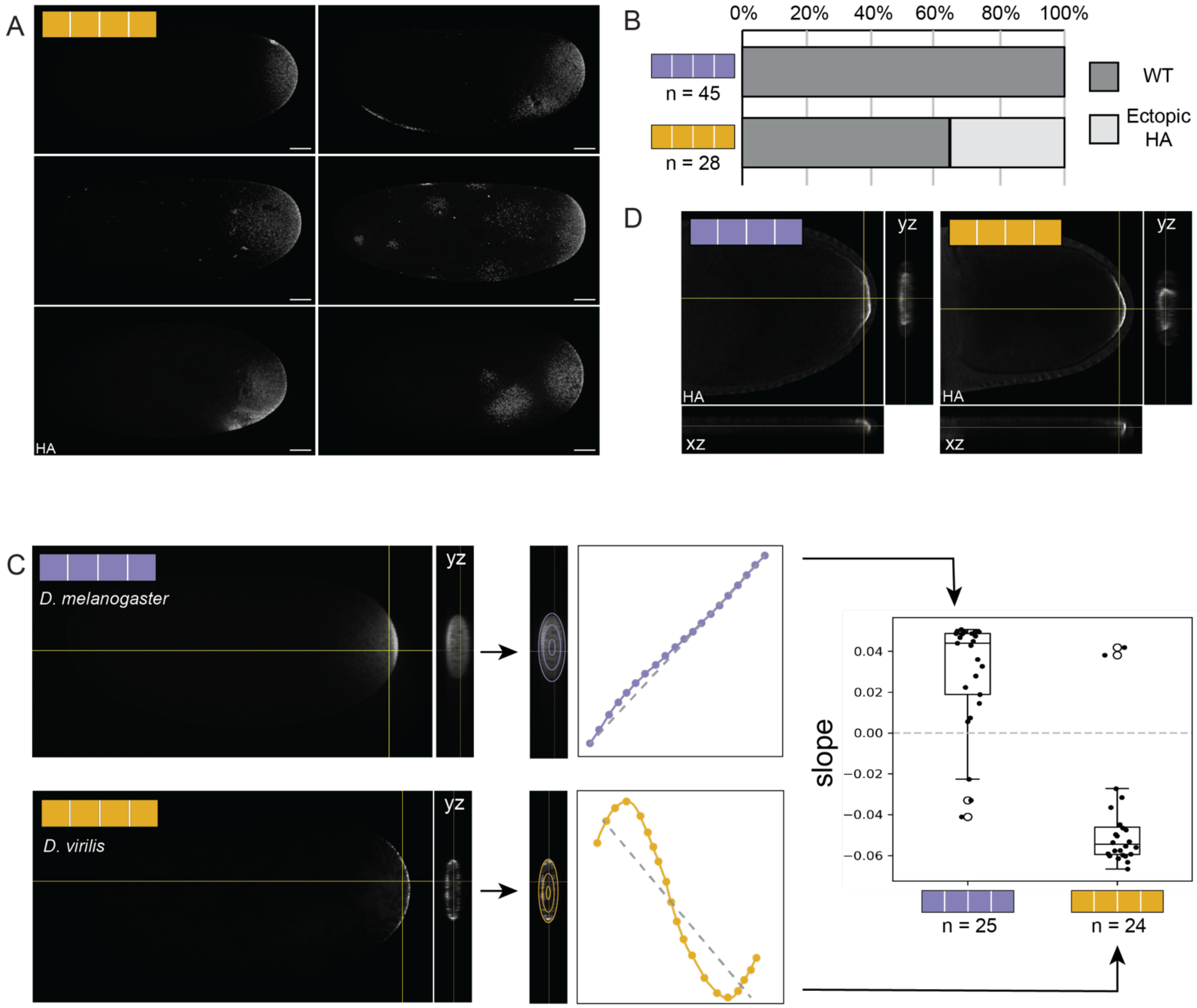

Figure S7. A) Cleavage stage embryos from *D. melanogaster* mothers expressing *D. virilis* Oskar exhibiting *D. virilis* Oskar localization patterns seen with anti-HA antibody stains. Micrographs are maximum intensity projections of 30 optical sections (step size = 2 μm), scale bars represent 50 μm and posterior is to the right. **B)** Frequency of ectopic patches of HA appearing away from the posterior pole in embryos from mothers expressing either *D. melanogaster* (left) or *D. virilis* (right) *oskar.* **C)** Schematic demonstrating how the plot in Fig. 6C was made. The yz projections from image stacks of anti-HA-stained embryos were eroded and fluorescent signal intensity measured 20 times. We drew a line of best fit for the curve of fluorescent signal intensity and plotted the slope of this line. **D)** Stage 10 oocytes from mothers expressing *D. melanogaster oskar* and *D. virilis oskar* stained with anti-HA antibody. Orthogonal views in the yz and xz orientations flank each image.

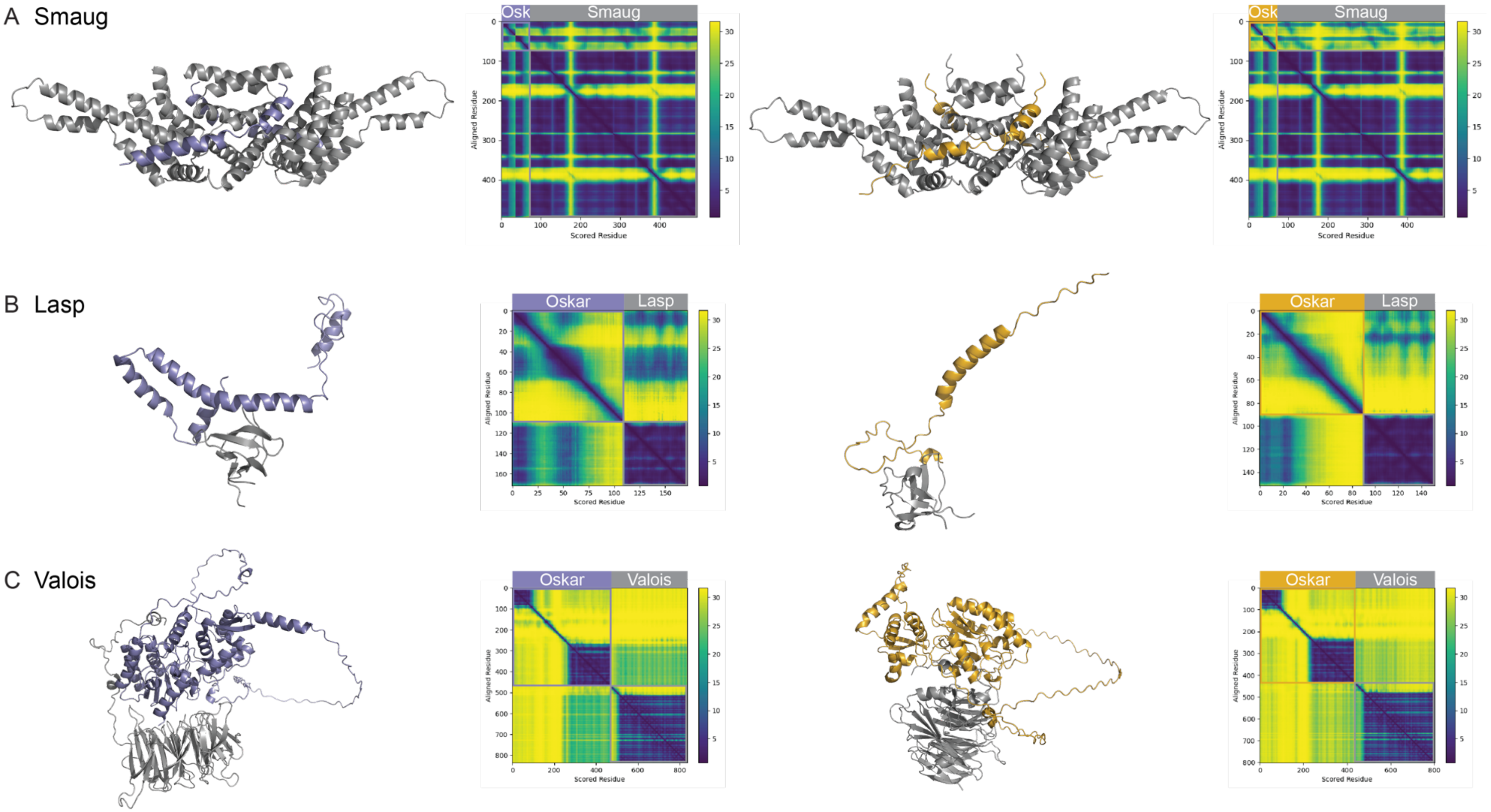

Figure S8. AlphaFold3 structural predictions and accompanying predicted aligned error plots for *D. melanogaster* Short Oskar (left) and *D. virilis* Short Oskar (right) with *D. melanogaster* Smaug (**A**), Lasp (**B**), and Valois (**C**). Only portions of Smaug [2], Lasp [3], and Valois [4] suggested by previous structural or *in vitro* assays to interact with Oskar, along with the corresponding portion of Oskar in this interaction, were used in the structural prediction.

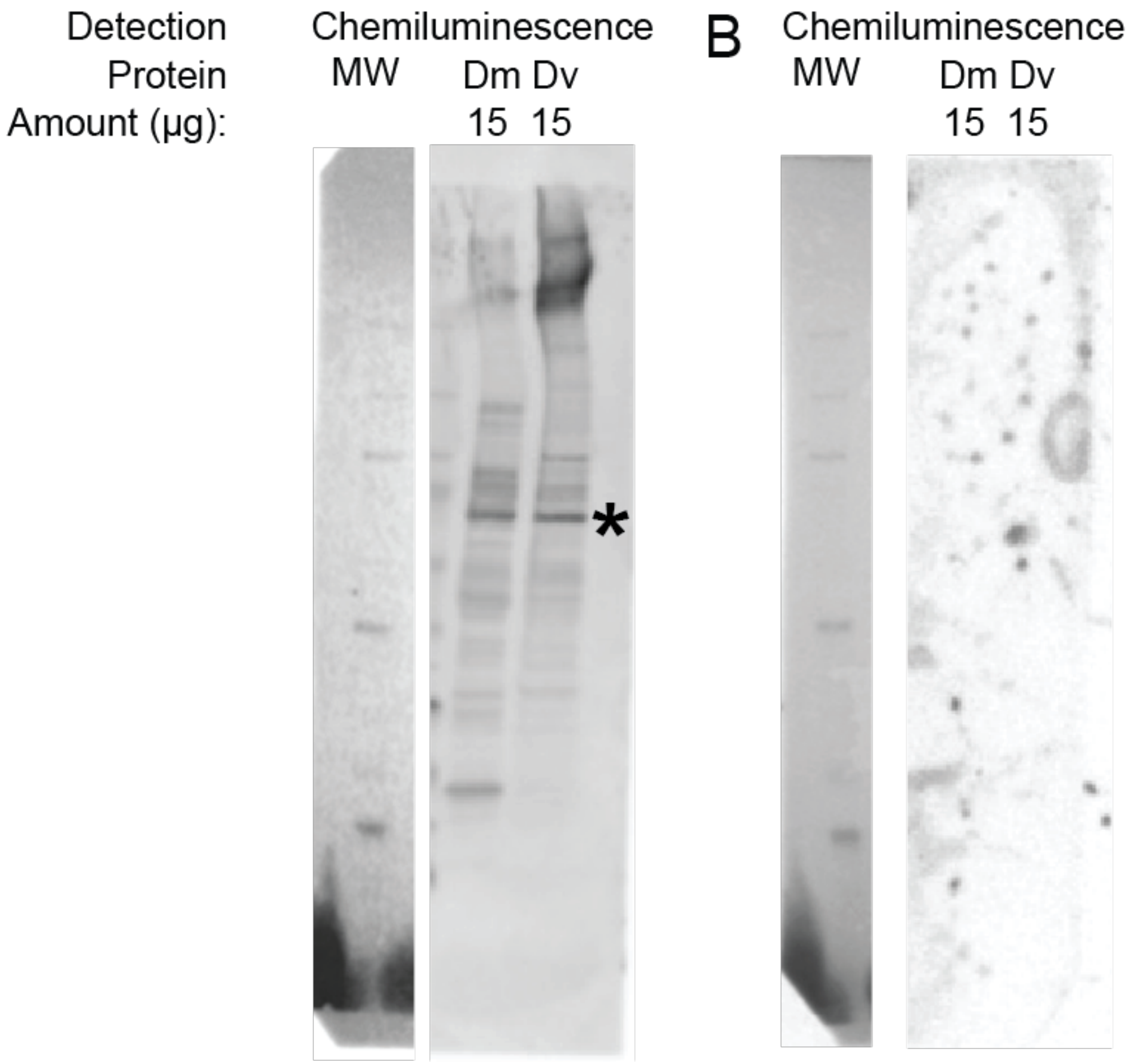

Figure S9. *D. virilis* anti-Oskar antibody validation. **A)** Chemiluminescent Western Blot with *D. melanogaster* (Dm) and *D. virilis* (Dv) ovary lysates incubated with anti-*Dvir* Oskar primary antibody. The amount of protein (μg) that was loaded for each lane is listed under the species identifier for each lane. Lane with molecular weight markers are marked MW. Bands at expected product sizes are marked with an asterisk (*Dmel* Oskar ~69 kDa, *Dvir* Oskar ~69 kDa). **B)** Control blot with no primary antibody. Note that this control experiment is the same one used to validate a second antibody that was generated at the same time for a different project[5] , and is reported as such in Figure S5 therein.

Pole cell count (*osk* null background)			
**Genotype**	**Sample size**	**μ**	**p-value**
Dm1234	37	22.22	n/a
Dv1234	21	0.00	0.00
Dm123Dv4	16	0.00	0.00
Dm12Dv34	15	1.13	0.00
Dm13Dv24	18	0.00	0.00
Dm23Dv14	20	1.55	0.00
Dm1Dv234	15	0.00	0.00
Dm3Dv124	21	0.00	0.00
Dm2Dv134	18	0.00	0.00
Dm4Dv123	21	25.19	0.04438
Dm14Dv23	23	30.61	6.49E-7
Dm34Dv12	32	9.88	4.14E-13
Dm234Dv1	21	9.33	0.00
Dm24Dv13	21	18.57	0.01105
Dm124Dv3	22	22.95	0.31977
Dm134Dv2	20	8.25	1.55E-15

Table S1. Statistical analysis of pole cell counts in stage 5 embryos expressing each Oskar chimera in the *oskar* RNA null background (*y[1] sc[*] v[1]; Kr[If-1]/CyO; TI{w[+mC]=TI}osk[0.EGFP]*). The p-values are estimated from the distribution of difference of means between sample and control from 100,000 simulated samples.

Germ plasm enrichment (*osk* null background)				
	**Genotype**	**Sample size**	**Integrated posterior enrichment p-value**	**Pearson correlation p-value**
Stage 10 oocyte: Vasa	Dm1234	49	n/a	
	Dv1234	43	0.20058	0.42069
	Dm123Dv4	63	6.38E-07	6.54E-12
	Dm12Dv34	26	6.33E-12	7.20e-13
	Dm13Dv24	116	0.00011	1.27e-08
	Dm23Dv14	75	1.55E-08	7.03e-09
	Dm1Dv234	77	8.99E-08	1.68e-05
	Dm3Dv124	101	0.0	8.17e-07
	Dm2Dv134	30	0.00121	0.00300
	Dm4Dv123	70	0.00141	6.92E-06
	Dm14Dv23	71	5.04E-11	0.08168
	Dm34Dv12	32	0.04972	0.00017
	Dm234Dv1	28	0.25847	0.00201
	Dm24Dv13	81	0.29487	0.00678
	Dm124Dv3	46	1.49E-06	0.23057
	Dm134Dv2	54	3.53E-10	0.39111
Stage 1–2 embryo: Vasa	Dm1234	54	n/a	
	Dv1234	26	1.07E-5	3.93E-13
	Dm123Dv4	32	0.03668	0.00014
	Dm12Dv34	11	0.12219	0.01178
	Dm13Dv24	35	0.00141	0.00
	Dm23Dv14	28	5.69E-5	7.67E-08
	Dm1Dv234	See table legend & Methods		
	Dm3Dv124	33	0.25939	0.00023
	Dm2Dv134	30	0.03191	0.00245
	Dm4Dv123	38	0.25513	8.31E-06
	Dm14Dv23	39	1.50E-8	0.00
	Dm34Dv12	56	0.12130	0.10232
	Dm234Dv1	24	0.49020	8.26E-7
	Dm24Dv13	32	0.10103	0.03315
	Dm124Dv3	35	0.07229	0.01482
	Dm134Dv2	35	0.41964	0.33832
Stage 1–2 embryo: *nanos*	Dm1234	60	n/a	

	Dv1234	30	0.17960	7.08E-14
	Dm123Dv4	20	0.00476	1.50E-09
	Dm12Dv34	9	2.01E-06	0.04266
	Dm13Dv24	25	0.00028	7.80E-25
	Dm23Dv14	23	0.14142	1.86E-15
	Dm1Dv234	See table legend & Methods		
	Dm3Dv124	39	3.40E-15	4.97E-34
	Dm2Dv134	30	0.24130	3.31E-19
	Dm4Dv123	37	0.15120	1.73E-06
	Dm14Dv23	32	0.00104	0.02030
	Dm34Dv12	32	0.00113	0.00112
	Dm234Dv1	42	8.97e-05	0.19292
	Dm24Dv13	22	0.39319	0.00110
	Dm124Dv3	47	7.80E-08	0.25014
	Dm134Dv2	33	1.73E-11	1.41E-07
Stage 1–2 embryo: *gcl*	Dm1234	56	n/a	
	Dv1234	24	0.03136	0.49283
	Dm123Dv4	29	0.07679	1.33E-11
	Dm12Dv34	9	0.00527	0.01495
	Dm13Dv24	33	0.44230	1.345E-05
	Dm23Dv14	21	0.03223	2.67E-14
	Dm1Dv234	See table legend & Methods		
	Dm3Dv124	46	0.06752	5.54E-21
	Dm2Dv134	21	0.00394	0.00545
	Dm4Dv123	37	0.00205	0.02295
	Dm14Dv23	36	0.00023	0.09993
	Dm34Dv12	23	0.08356	0.02337
	Dm234Dv1	59	0.00136	7.82E-11
	Dm24Dv13	25	0.14451	0.14037
	Dm124Dv3	36	0.06186	0.47409
	Dm134Dv2	74	0.19011	0.01637

Table S2. Statistical analysis of Vasa, *nanos*, and *gcl* posterior enrichment and Pearson correlation with HA in stage 10 oocytes and stage 1–2 embryos expressing each Oskar chimera in the *oskar* RNA null background *w[1118]; Kr[If-1]/CyO; TI{w[+mC]=TI}osk[0.EGFP]*. For the chimeric genotype with the Long Osk domain from *D. melanogaster* and the other three domains from *D. virilis* (Dm1Dv234), sufficient posterior HA enrichment was found in oocytes to pass our threshold for analysis of germ plasm enrichment, but no embryos had sufficient enrichment to be included in the analysis.

P-values are estimated from the distribution of difference of means between sample and control from 100,000 simulated samples.

**Pole cell count and axial patterning (endog. *osk* background)**							
Genotype	Stage 5 embryo: Pole cell counts				1st instar larva: Cuticle patterning		
	Sample size	μ	With negative control p-value	With positive control p-value	Sample size	JSD negative	JSD positive
Dm1234	21	62.05	5.99E-18	n/a	46	0	n/a
Dv1234	21	27.86	1.95E-7	0.00	31	1	1
Dm123Dv4	20	37.15	0.04990	2.10E-15	60	0.04297	0.04297
Dm12Dv34	26	35.23	0.01277	0.00	53	0.00950	0.00950
Dm13Dv24	20	3.80	0.00	0.00	51	1	1
Dm23Dv14	20	49.80	0.00330	1.72E-5	47	0.09083	0.09083
Dm1Dv234	20	40.70	0.28557	2.77E-15	51	0.04037	0.04037
Dm3Dv124	20	37.80	0.06714	7.77E-16	45	0	0
Dm2Dv134	20	45.35	0.17532	1.25E-6	54	0.55964	0.55964
Dm4Dv123	20	51.55	0.00020	0.00010	53	0.00950	0.00950
Dm14Dv23	20	59.30	1.41E-9	0.18236	57	0.01777	0.01777
Dm34Dv12	30	57.03	3.47E-13	0.01352	50	0	0
Dm234Dv1	22	46.68	0.03862	8.50E-9	52	0.04983	0.04983
Dm24Dv13	20	64.30	1.39E-20	0.19133	51	0	0
Dm124Dv3	33	44.42	0.15442	1.80E-13	60	0.06094	0.06094
Dm134Dv2	20	38.30	0.07193	0.00	57	0.02683	0.02683
endog. *osk* only	34	42.15	n/a	0.00	55	n/a	0

Table S3. Left: Statistical analysis of pole cell counts in stage 5 embryos expressing one copy of each Oskar chimera in a background with endogenous *oskar* present. Right: Jensen–Shannon Divergence (JSD) calculated for the distribution of “WT,” “Strong AP Defects,” and “Partial AP Defects” phenotypes in each chimeric background compared to the distribution of these phenotypes in the negative (endogenous *oskar* only) and positive control (Dm1234).

Germ plasm enrichment (endog. *osk* background)				
	Genotype	Sample size	With negative control p-value	With positive control p-value
Stage 10 oocyte: Vasa	Dm1234	52	1.48E-10	n/a
	Dv1234	53	4.10E-21	0.11051
	Dm123Dv4	64	6.46E-15	0.37868
	Dm12Dv34	39	6.55E-8	0.20238
	Dm13Dv24	67	0.03216	3.17E-5
	Dm23Dv14	42	4.17E-7	0.07693
	Dm1Dv234	41	2.58E-18	0.08042
	Dm3Dv124	65	4.96E-7	0.14429
	Dm2Dv134	37	1.86E-31	0.00423
	Dm4Dv123	74	1.36E-5	0.00587
	Dm14Dv23	66	0.00073	0.00093
	Dm34Dv12	37	4.15E-5	0.10557
	Dm234Dv1	32	4.68E-6	0.01924
	Dm24Dv13	51	2.25E-8	0.11377
	Dm124Dv3	61	1.78E-9	0.10925
	Dm134Dv2	40	1.34E-22	0.03775
	endog. *osk* only	179	n/a	1.36E-10
Stage 1–2 embryo: Vasa	Dm1234	125	0.00056	n/a
	Dv1234	16	1.47E-6	0.00238
	Dm123Dv4	73	8.02e-9	0.00131
	Dm12Dv34	37	0.31201	0.01605
	Dm13Dv24	58	0.00016	0.20395
	Dm23Dv14	56	0.02356	0.21459
	Dm1Dv234	31	0.29723	0.03034
	Dm3Dv124	82	0.00159	0.44346
	Dm2Dv134	23	0.00578	0.24946
	Dm4Dv123	134	1.36e-5	0.15072
	Dm14Dv23	54	0.24605	0.00250
	Dm34Dv12	60	0.03906	0.15357
	Dm234Dv1	37	0.21932	0.02844
	Dm24Dv13	38	0.36906	0.00322
	Dm124Dv3	88	0.30718	9.40E-6
	Dm134Dv2	132	0.43041	1.13e-5
	endog. *osk* only	56	n/a	0.00052
	Dm1234	49	4.03e-7	n/a

Stage 1–2 embryo: *nanos*	Dv1234	31	4.44E-16	0.00
	Dm123Dv4	91	0.49110	2.66E-6
	Dm12Dv34	36	0.43363	0.00062
	Dm13Dv24	48	0.00	0.00
	Dm23Dv14	71	0.0927	0.00041
	Dm1Dv234	27	0.26759	0.00028
	Dm3Dv124	30	0.00459	2.84E-8
	Dm2Dv134	28	7.89E-7	1.28E-14
	Dm4Dv123	30	0.00275	0.18410
	Dm14Dv23	104	5.80E-7	0.29581
	Dm34Dv12	30	0.00017	0.39860
	Dm234Dv1	23	0.00167	0.23828
	Dm24Dv13	58	2.34E-5	0.13648
	Dm124Dv3	50	0.17035	0.00110
	Dm134Dv2	49	0.21680	6.42E-6
	endog. *osk* only	133	n/a	3.78E-7
Stage 1–2 embryo: *pgc*	Dm1234	49	1.38E-18	n/a
	Dv1234	31	4.24E-8	0.00
	Dm123Dv4	91	0.01503	1.04E-12
	Dm12Dv34	36	0.01208	1.62e-8
	Dm13Dv24	48	3.42E-6	0.00
	Dm23Dv14	71	1.13E-13	0.00
	Dm1Dv234	27	0.09671	6.85E-10
	Dm3Dv124	30	0.11079	5.83E-14
	Dm2Dv134	28	1.97E-5	0.00
	Dm4Dv123	30	1.09E-7	2.35E-5
	Dm14Dv23	104	6.03E-9	1.12E-8
	Dm34Dv12	30	5.70E-13	0.06107
	Dm234Dv1	23	0.00412	1.27E-5
	Dm24Dv13	58	4.75E-8	1.54E-5
	Dm124Dv3	50	2.55E-11	0.00144
	Dm134Dv2	49	2.76E-6	4.26E-6
	endog. *osk* only	133	n/a	0.00

Table S4. Statistical analysis of Vasa, *nanos,* and *pgc* enrichment in stage 10 *D. melanogaster* oocytes and stage 1–2 embryos expressing each Oskar chimera in a background with endogenous *oskar* present.

Radial Oskar localization	
**Genotype**	**Sample size**
D. mel osk null + *D. mel osk* transgene	25
D. mel osk null + *D. vir osk* transgene	24
D. melanogaster (Oregon R)	25
D. virilis	56
D. mel osk null + *Dm123Dv4* transgene	34
	**p-value**
D. mel osk null + *D. mel osk* transgene vs. *D. mel osk* null + *D. vir osk* transgene	1.21E-15
D. mel osk null + *D. mel osk* transgene vs. *D. melanogaster* (Oregon R)	0.05499
D. mel osk null + *D. mel osk* transgene vs. *D. virilis*	0.00103
D. mel osk null + *D. mel osk* transgene vs. *D. mel osk* null + *Dm123Dv4 osk* transgene	0.30309
D. mel osk null + *D. vir osk* transgene vs. *D. melanogaster* (Oregon R)	0.00
D. mel osk null + *D. vir osk* transgene vs. *D. virilis*	4.71E-8
D. mel osk null + *D. vir osk* transgene vs. *D. mel osk* null + *Dm123Dv4 osk* transgene	0.00
D. melanogaster (Oregon R) vs. *D. virilis*	2.40E-09
D. melanogaster (Oregon R) vs. *D. mel osk* null + *Dm123Dv4 osk* transgene	0.00202
D. virilis vs.*D. mel osk* null + *Dm123Dv4 osk* transgene	0.00127

Table S5. Statistical analysis of radial HA localization in cleavage stage embryos. The p-values are estimated from the distribution of difference of means between sample and control from 100,000 simulated samples.

**Hybridization Chain Reaction Probes**			
**Species**	**Probe**	**Amplifier**	**Fluorophore**
D. melanogaster	*oskar*	B1	Alexa 488
	Nanos	B5	Alexa 555
	germ cell-less	B3	Alexa 594
	polar granule component	B4	Alexa 647
D. virilis	*oskar*	B1	Alexa 488
	nanos	B5	Alexa 555
	germ cell-less	B3	Alexa 594
	polar granule component	B4	Alexa 647

Table S7. List of hybridization chain reaction (HCR) probes used in fluorescence *in situ* hybridization experiments.

Primer sequences	
**Name**	**Sequence**
pVAL22_gOsk_bridge2_R	CACCCTCAGCTCCAAAGCCTCGAGTGTTTTGCCATTTTACA
pVAL22_gOsk_bridge1_R	CACTAGTAGTACCAGCTTATAACTTCGTATAATGTGAATTGGGTACCGGGCC
pVAL22_gOsk_bridge2_F	TGTAAAATGGCAAAACACTCGAGGCTTTGGAGCTGAGGGTG
pVAL22_gOsk_bridge1_F	GGCCCGGTACCCAATTCACATTATACGAAGTTATAAGCTGGTACTACTAGTG
pVALIUM22_R6	GCTTTGGAGCTGAGGGTG
pVALIUM22_F6	ACATTATACGAAGTTATAAGCTGGTACTACTAGTG
Dmel_gOsk_HA_F1	GATGTTCCGGATTACGCTTAAGTTGGGTTCTTAATCAAGATACATATATG
Dmel_gOsk_HA_R1	GTATGGGTAACTACCATACTCCAGACTCGTTTCAATAACTTGC
HA_F1	GGTAGTTACCCATACGATGTTCCGGATTACGCT
Dmel_Dvir4_R1	TAATCCGGAACATCGTATGGGTAACTACCATAATACTCCAAGTTTTCTTCAATCATGTGC
Dmel_upstream_F1	CGCTTGGAGGAAAGTGATCCAATATTCTTG
Dmel_Dvir1_F1	GAATATTGGATCACTTTCCTCCAAGCGATGGCGACATTCAGAAGTGAATTC
Dmel_upstream_F2	CTGTTGAATTCACTTCTGAATGTCGCCATCGCTTGGAGGAAAGTGATCCAATATTCTTG
Dmel_Dvir1_F2	ATGGCGACATTCAGAAGTGAATTCAACAGCGTGCCGAACACTTACAACC
Dvir_Losk_F161	ATGACCATCATGGACAACACCTATATT
Dvir_int_R1	CCAGCAGTATGGAGCGTATTTCAG
Dvir_Losk_F261	GACAGTGTGAGCAACCCAATC
Dvir_Losk_F401	TCGGACTATGATGCATATCTATTGG
Dvir_Losk_F387	TCGTGTCCATCAACGCAAC
Dvir_int_F1	GTACATGGTTATGATTTCGAGGCT
Dvir_int_F2	CAAATCAACCGACTCCATTCCC
Dmel_gOsk_insert_F1	AACTGCTCACCGTTACTTCTGTTG
Dmel_Losk_R414	GTTAATCGTCAGCAGAGAATCGTTG
Dmel_Losk_R400	TAAAATCGTTGGCGTGGGC
Dmel_Losk_F140	ACCATCATCGAGAGCAACTATATATCCG

Dmel_Losk_gOsk_R1	CATCGCTTGGAGGAAAGTGATC
Dmel_Dvir4_R2	CCGGAACATCGTATGGGTAACTACCATAATACTCCAAGTTTTCTTCAATCATGTGC
Dmel_upstream_F3	GAATTCACTTCTGAATGTCGCCATCGCTTGGAGGAAAGTGATCCAATATTCTTG
Dmel_Dvir1_F2	ATGGCGACATTCAGAAGTGAATTCAACAGCGTGCCGAACACTTACAACC
Dvir_Losk_R386	CAACGTTGTTGGCGTGCGCG
Dm4_Dv3_F1	CGCGCACGCCAACAACGTTGACCAGTGGAACCTACAACGATTCTC
Dm4_HA_R1	CCGGAACATCGTATGGGTAACTACCATACTCCAGACTCGTTTCAATAACTTGC
Dvir_Losk_R260	GCGTTGTCGCTGCTGCAG
Dm3_Dv2_F1	TACTGCAGCAGCGACAACGCTACAGCAGCGGAGCTCCG
Dm3_Dv4_R1	TGTTGCGTTGATGGACACGATAAAATCGTTGGCGTGGGC
Dm2_Dv1_F1	GTGCAAGTATCACCAGCTCCAGCATGACCATCATCGAGAGCAACTATATATC
Dvir_Losk_R160	GCTGGAGCTGGTGATACTTGCAC
Dm2_Dv3_R1	TGGATTGGGTTGCTCACACTGTCGTCGCTGGTGCGCTCTTTC
Dm1_Dv2_R1	CCAATATAGGTGTTGTCCATGATGGTCATGTTGTTCTCGCTGGTGTTGCTG
Dm1_gOsk_F1	GAATATTGGATCACTTTCCTCCAAGCGATGGCCGCAGTCACAAGTG

Table S8. Primer sequences used for cloning *oskar* chimeras.

Supplemental References

1. Abramson, J. *et al.* Accurate structure prediction of biomolecular interactions with AlphaFold 3. *Nature* **630**, 493–500 (2024).

2. Kubíková, J., Ubartaitė, G., Metz, J. & Jeske, M. Structural basis for binding of Drosophila Smaug to the GPCR Smoothened and to the germline inducer Oskar. *Proc. Natl. Acad. Sci.* **120**, e2304385120 (2023).

3. Suyama, R., Jenny, A., Curado, S., Berkel, W. P. & Ephrussi, A. The actin-binding protein Lasp promotes Oskar accumulation at the posterior pole of the Drosophila embryo. *Development* **136**, 95–105 (2009).

4. Anne, J. Targeting and Anchoring Tudor in the Pole Plasm of the Drosophila Oocyte. *PLoS ONE* **5**, e14362 (2010).

5. Repouliou, A., Srouji, J. R., Rivard, E. L., Leschziner, A. E. & Extavour, C. G. The LOTUS domain of Oskar promotes localisation of both protein and mRNA components of Drosophila germ plasm. *bioRxiv* 2025.05.02.651258 (2025) doi:10.1101/2025.05.02.651258.
